# Resistant Hypertension: Integration of Novel Agents and Interventional Approaches in Clinical Practice

**DOI:** 10.31083/RCM45429

**Published:** 2026-01-22

**Authors:** Jose Arriola-Montenegro, Andres Chaponan-Lavalle, Natalia Nombera-Aznaran, Mohammed Abdalla, Benjamin Bizer, Arjunmohan Mohan, Irma Andrea Muñoz Verdugo, Pooya Zardoost, Karina Ordaya-Gonzales, Alan Villarreal Rizzo, Wagner Rios-Garcia, Laverne Kar Yin Yip, Maria L. Gonzalez Suarez

**Affiliations:** ^1^Division of Nephrology and Hypertension, Mayo Clinic, Rochester, MN 55905, USA; ^2^Department of Medicine, Universidad Peruana de Ciencias Aplicadas, 15023 Lima, Peru; ^3^Department of Nephrology, University of Alabama at Birmingham, Birmingham, AL 35294, USA; ^4^Division of Internal Medicine, Mayo Clinic, Rochester, MN 55902, USA; ^5^Faculty of Human Medicine, National University of San Luis Gonzaga, 11004 Ica, Peru

**Keywords:** resistant hypertension, secondary hypertension, mineralocorticoid receptor antagonists, renal denervation, SGLT2 inhibitors, precision medicine, artificial intelligence, therapeutic algorithms

## Abstract

Resistant hypertension (RH) is a high-risk phenotype characterized by blood pressure readings ≥130/80 mmHg despite maximally tolerated therapy with three antihypertensive agents, including a diuretic, or controlled blood pressure requiring four or more medications. The diagnosis of RH requires a structured evaluation that confirms accurate blood pressure measurement, excludes pseudoresistance—particularly nonadherence and white coat hypertension—and identifies secondary causes such as obstructive sleep apnea, primary aldosteronism, renovascular disease, pheochromocytoma, and Cushing syndrome. RH arises from overlapping mechanisms, including activation of the renin–angiotensin–aldosterone system (RAAS), sympathetic overactivity, arterial stiffness, volume expansion, and immune-mediated pathways. Management begins with lifestyle modification and optimized triple therapy, followed by mineralocorticoid receptor antagonists as the preferred fourth-line treatment. Emerging pharmacological options, such as sodium–glucose cotransporter-2 (SGLT2) inhibitors, glucagon-like peptide-1 (GLP-1) receptor agonists, endothelin receptor antagonists, aldosterone synthase inhibitors, and angiotensin receptor and neprilysin inhibitors (ARNIs), offer additional therapeutic potential; meanwhile, device-based interventions, including renal denervation and baroreflex activation therapy, have shown sustained blood pressure reductions in selected patients. Future directions highlight precision medicine, digital health technologies, and artificial intelligence as methods to improve diagnosis, guide individualized therapy, and enhance long-term blood pressure control.

## 1. Introduction

Hypertension is the most common modifiable cardiovascular risk factor and a leading cause of morbidity and mortality worldwide [1]. Resistant hypertension (RH) represents the most severe phenotype and is strongly associated with hypertension-related organ damage, as well as increased cardiovascular and renal morbidity and mortality. RH is defined as blood pressure (BP) ≥130/80 mmHg that remains above target despite concurrent use of three antihypertensive agents from different classes, including a diuretic, all at optimal or maximally tolerated doses and after ensuring adherence to recommended lifestyle modifications, such as dietary sodium restriction, weight loss, physical activity, and moderation of alcohol intake [2, 3, 4]. Patients with controlled BP on four or more medications are classified as having “controlled resistant hypertension” [2], while “refractory hypertension” refers to a rare subset in which BP remains uncontrolled despite the use of five or more antihypertensive drugs [5].

The true prevalence of RH is difficult to determine due to frequent misclassification from suboptimal treatment, non-adherence, and the white coat effect. A 2019 meta-analysis of 91 studies estimated the prevalence of apparent RH at 14.7% among treated hypertensive patients, which decreased to 10.3% after excluding confounders [6]. Prevalence is even higher in high-risk populations, such as those with chronic kidney disease (CKD) (22.9%) or heart failure (HF) (13.7%) [6]. These findings emphasize the importance of accurate diagnosis and targeted evaluation in selected groups.

This review provides an overview of RH, with emphasis on diagnostic strategies, pathophysiology, evaluation, and management. We highlight current treatment strategies, with particular emphasis on emerging pharmacologic therapies and interventional approaches.

## 2. Search Strategy

A comprehensive literature search was conducted to identify relevant publications addressing resistant hypertension, its pathophysiology, pharmacological management, and interventional strategies. The databases PubMed/MEDLINE, Embase, Scopus, and the Cochrane Library were systematically searched for studies published between January 2000 and September 2025. The search strategy combined Medical Subject Headings (MeSH) and free-text terms, including: “resistant hypertension”, “refractory hypertension”, “uncontrolled blood pressure”, “antihypertensive therapy”, “renal denervation”, “baroreflex activation”, “sodium–glucose cotransporter-2 (SGLT2) inhibitors”, “GLP-1 receptor agonists”, and “device-based therapies”.

References from major guidelines, including the 2024 ESC Guidelines, 2025 AHA/ACC Guidelines, 2020 ISH Global Guidelines, 2021 KDIGO Blood Pressure Guidelines, and 2023/2024 ESH Practice Guidelines, were also reviewed to ensure completeness. Inclusion criteria comprised clinical trials, meta-analyses, observational studies, and major society guidelines related to resistant hypertension in adults. Case reports, editorials, pediatric studies, and non-English publications were excluded. The screening process adhered to the PRISMA 2020 framework to ensure transparency and reproducibility, although the present work was designed as a narrative review rather than a formal systematic review.

## 3. Diagnosis and Evaluation

**Fig. 1.**
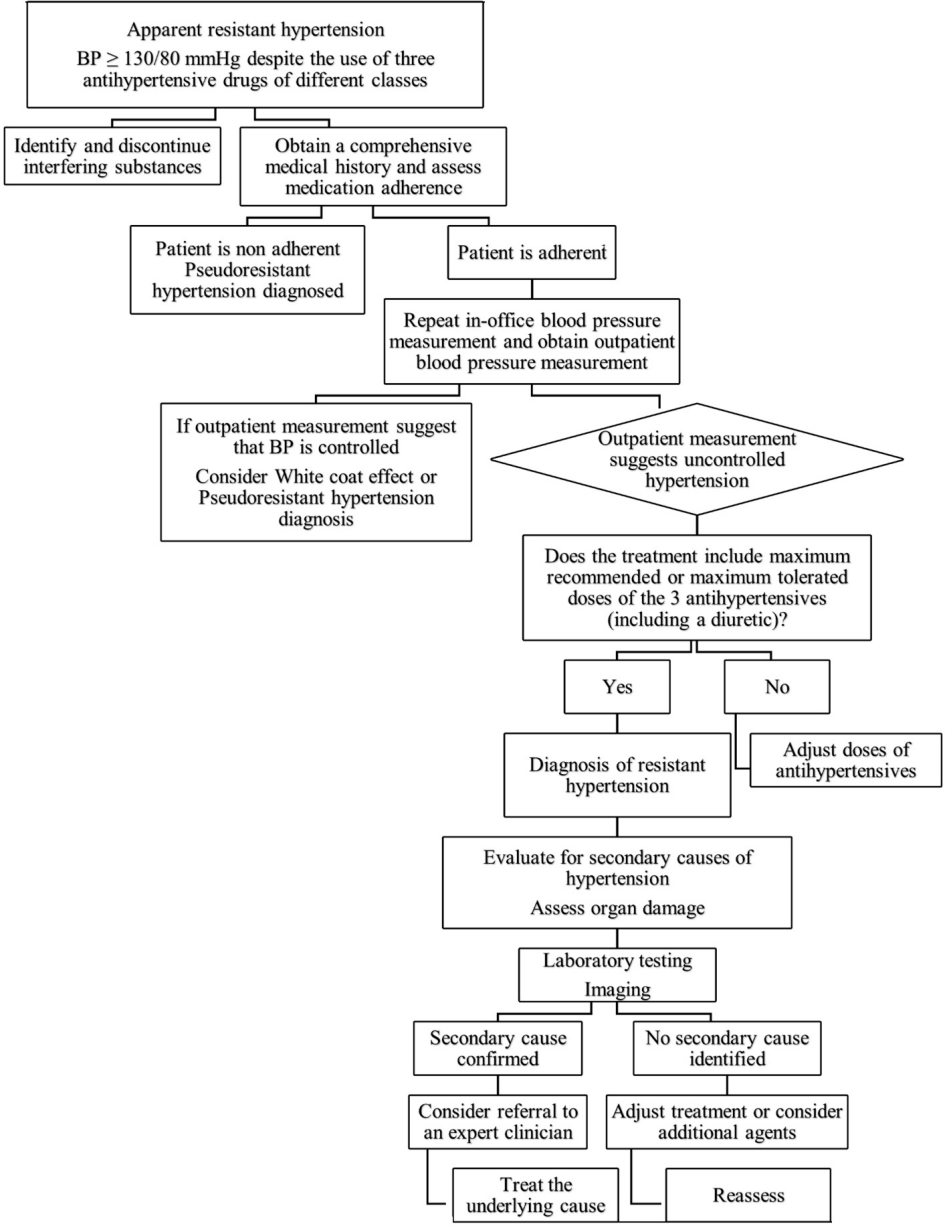
**Stepwise evaluation and management of apparent resistant hypertension**. This algorithm summarizes the recommended approach to patients with blood pressure ≥130/80 mmHg despite using three antihypertensive agents. It emphasizes confirming adherence, ruling out pseudoresistance and white-coat effect, verifying adequate medication dosing, and assessing for secondary hypertension. Subsequent steps include targeted laboratory and imaging evaluation, specialist referral when appropriate, and treatment adjustments based on identified causes. © “Used with permission of Mayo Foundation for Medical Education and Research, all rights reserved.”

Accurate diagnosis of RH requires a systematic and stepwise approach that incorporates standardized blood pressure measurement techniques, out-of-office confirmation, and comprehensive assessment of contributing factors. Major clinical guidelines, including those from the National Heart Foundation of Australia (2016) [7], the American Heart Association (2018) [2], the European Society of Hypertension (2024) [8, 9], Hypertension Canada (2020) [10], and the International Society of Hypertension (2020) [11], highlight the importance of confirming elevated BP using validated protocols before diagnosing RH. The process begins with proper office-based measurement, ideally using automated office BP (AOBP) to reduce observer bias and improve reproducibility [12]. Ambulatory (ABPM) or home BP monitoring is essential to exclude white coat hypertension, which may be present in up to 20–30% of patients with apparent RH [13, 14]. A critical step is the evaluation of adherence, given that non-adherence is the leading cause of pseudoresistance [15, 16]. Assessment should include structured interviews, pill counts, pharmacy refill data, and, when available, therapeutic drug monitoring or biochemical validation [17, 18]. Once white coat effect and non-adherence are ruled out, further evaluation includes identifying secondary causes of hypertension, assessing target organ damage, and ensuring optimization of pharmacologic therapy [2, 19]. This algorithmic approach (Fig. 1) helps differentiate true RH from other entities and guides clinicians toward appropriate management strategies.

## 4. Pathophysiology of Resistant Hypertension

**Fig. 2.**
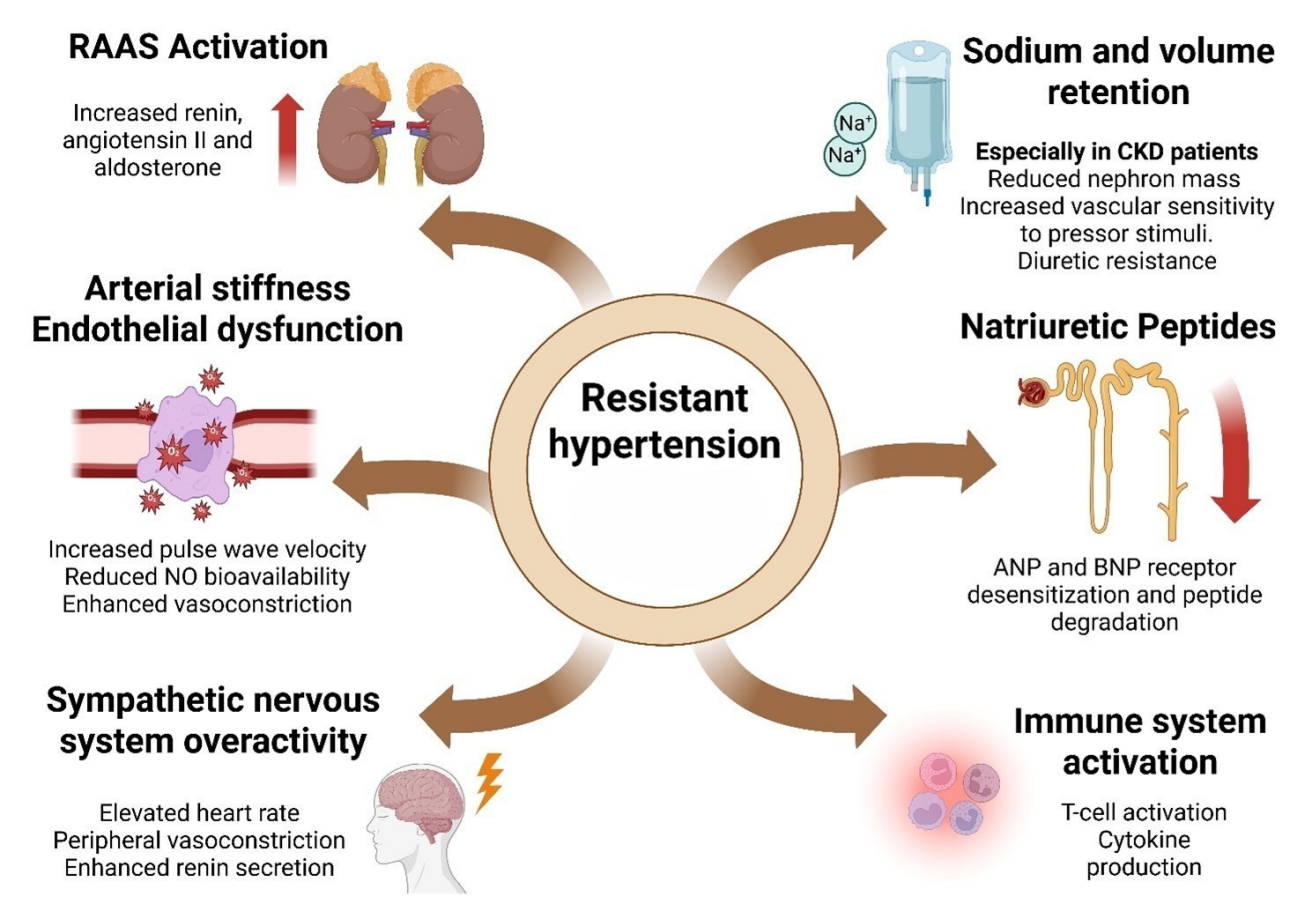
**Pathophysiological mechanisms driving resistant hypertension**. The figure summarizes major mechanisms underlying resistant hypertension. These include activation of the renin–angiotensin–aldosterone system (RAAS); arterial stiffness and endothelial dysfunction; sympathetic nervous system (SNS) overactivity; sodium and volume retention, particularly in chronic kidney disease (CKD); impaired natriuretic peptide (ANP/BNP) signaling; and immune system activation. Together, these pathways promote persistent hypertension despite optimal therapy. Created with BioRender.com (https://www.biorender.com) (License ID: PC28J3W2T9). © 2025 BioRender. “Used with permission of Mayo Foundation for Medical Education and Research, all rights reserved.”

RH is a complex and multifactorial condition. The pathophysiology involves an interplay of neurohormonal, renal, vascular, and immunologic mechanisms that lead to persistent elevation of systemic vascular resistance and volume overload (Fig. 2).

### 4.1 Activation of the Renin–Angiotensin–Aldosterone System (RAAS)

Overactivation of the RAAS plays a central role in RH. Increased renin release stimulates angiotensin II production, a potent vasoconstrictor that also promotes aldosterone secretion. Aldosterone contributes to sodium and water retention, vascular inflammation, and remodeling, perpetuating elevated BP. In RH, RAAS activation may be inappropriate or resistant to negative feedback, contributing to pharmacologic resistance [20].

### 4.2 Sodium and Volume Retention (Especially in CKD)

Volume overload, often due to impaired natriuresis, is a key contributor to RH. This is especially pronounced in patients with CKD, where reduced nephron mass impairs sodium excretion, leading to extracellular fluid expansion and elevated BP. Even in the absence of overt fluid overload, sodium retention sensitizes the vasculature to pressor stimuli. Diuretic resistance may further exacerbate volume-dependent hypertension in this population [21].

### 4.3 Natriuretic Peptides

Although natriuretic peptides such as ANP and BNP promote vasodilation and natriuresis, their compensatory effects are often insufficient in RH. In advanced disease states, receptor desensitization or peptide degradation limits their protective effects. Reduced natriuretic peptide activity may contribute to volume expansion and impaired vascular compliance [22].

### 4.4 Sympathetic Nervous System (SNS) Overactivity

Increased SNS activity is another major driver of RH. It contributes to elevated heart rate, peripheral vasoconstriction, and enhanced renin secretion. Factors such as obesity, obstructive sleep apnea, and stress can chronically stimulate the SNS, leading to sustained hypertension and reduced baroreceptor sensitivity [23].

### 4.5 Arterial Stiffness and Endothelial Dysfunction

With aging, diabetes, and chronic hypertension, arterial stiffness increases, impairing the Windkessel effect and leading to elevated systolic BP and widened pulse pressure. In RH, increased pulse wave velocity and endothelial dysfunction reduce nitric oxide bioavailability, enhance vasoconstriction, and promote vascular inflammation. This contributes to a maladaptive vascular response to standard antihypertensive therapy [24].

### 4.6 Immune System Activation

Emerging evidence highlights the role of innate and adaptive immunity in the pathogenesis of RH. T-cell activation and cytokine production promote renal sodium retention, oxidative stress, and vascular dysfunction. Immune-mediated mechanisms may underlie the limited response to conventional pharmacotherapy in some RH patients [25].

## 5. Screening for Secondary Causes of Hypertension

### 5.1 Obstructive Sleep Apnea

Obstructive sleep apnea (OSA) is the most prevalent secondary cause of hypertension, affecting 25 to 50% of individuals with elevated blood pressure [3]. Recurrent episodes of hypoxia and hypercapnia during sleep activate the sympathetic nervous system and RAAS, contributing to sustained hypertension. Screening for OSA should be prioritized in patients with suggestive symptoms such as daytime somnolence or loud snoring, and in those with risk factors including advanced age, male sex, obesity, and increased neck circumference [26]. The STOP-BANG questionnaire is a practical tool to estimate OSA risk based on clinical features [27], while overnight oximetry can further support screening, especially when used in combination with STOP-BANG [28]. However, the diagnostic gold standard remains overnight polysomnography, which quantifies disease severity using the Apnea-Hypopnea Index (AHI) [29]. First-line treatment is continuous positive airway pressure (CPAP), which, when used for ≥5 hours per night, has been shown to reduce blood pressure in hypertensive patients with OSA [3, 30].

### 5.2 Primary Aldosteronism

Current clinical guidelines recommend screening for primary aldosteronism using the plasma aldosterone-to-renin activity ratio (ARR) in patients with RH [31, 32]. This remains essential even in the absence of hypokalemia or metabolic alkalosis, as hypokalemia—traditionally considered a hallmark finding—is present in fewer than 20% of cases. Recent studies employing ARR have revealed a higher prevalence of primary aldosteronism than previously recognized when relying solely on hypokalemia as a diagnostic marker. Early detection enables targeted treatment with mineralocorticoid receptor antagonists or surgical intervention, significantly improving blood pressure control and reducing cardiovascular risk in patients with refractory hypertension [33, 34, 35].

### 5.3 Renovascular Hypertension

Renovascular hypertension results from renal artery stenosis, which reduces renal perfusion and activates the renin-angiotensin-aldosterone system, leading to vasoconstriction and elevated blood pressure [36]. If left untreated, parenchymal kidney damage may develop unilaterally or bilaterally. The two most common causes are atherosclerotic renovascular disease—typically seen in older adults—and fibromuscular dysplasia, which primarily affects women under 40 years of age [37]. Atherosclerotic disease often coexists with coronary, cerebrovascular, or peripheral arterial disease. Clinical suspicion for renovascular hypertension should be high in patients presenting with recurrent flash pulmonary edema, acute kidney injury following angiotensin-converting enzyme inhibitors (ACEis) or angiotensin receptor blockers (ARBs) initiation, or asymmetric kidney sizes on imaging [38]. In contrast, fibromuscular dysplasia should be considered in young women (age 35–40) with sudden-onset hypertension and no significant medical history [39]. Diagnostic imaging modalities such as duplex Doppler ultrasound, magnetic resonance angiography (MRA), and computed tomographic angiography (CTA) are effective for detecting renal artery stenosis [40]. Initial management is typically medical, using antihypertensive agents. Revascularization with percutaneous transluminal angioplasty is generally reserved for selected cases, such as refractory hypertension, arterial occlusion, or failed medical therapy. Three major randomized controlled trials (STAR, ASTRAL, and CORAL) did not demonstrate a significant benefit of angioplasty over medical therapy in most patients [41]. However, patients with recurrent flash pulmonary edema, unexplained HF, or progressive renal dysfunction due to bilateral disease may benefit from revascularization [42]. In contrast, those with advanced CKD or proteinuria >1000 mg/24 h are less likely to respond [43]. Patients with fibromuscular dysplasia often show favorable outcomes with angioplasty [44].

### 5.4 Pheochromocytoma 

Pheochromocytoma is a catecholamine-secreting tumor arising from chromaffin cells of the adrenal medulla, producing norepinephrine, epinephrine, or dopamine. Hypertension may be sustained or paroxysmal. Fewer than 50% of patients present with the classic triad: episodic headache, diaphoresis, and hypertension [45]. Diagnostic evaluation is indicated in cases of: resistant hypertension (BP ≥130/80 mmHg on three antihypertensives including a diuretic), incidentally discovered adrenal masses with >10 Hounsfield units on computed tomography scans, or hypertensive crises triggered by anesthesia or surgery [46, 47, 48]. Biochemical testing includes plasma fractionated metanephrines (sensitivity 91%, specificity 99%) [49] and 24-hour urine fractionated metanephrines (sensitivity 92%, specificity 89%) [50]. Imaging should follow biochemical confirmation and typically begins with computed tomography scans. For metastatic or advanced disease, gallium-68 DOTATATE-PET, fluorodeoxyglucose-PET, iodine-metaiodebenzoglunidine, or octreotide scanning can also be used [51]. Definitive treatment is surgical resection [52]. When surgery is not feasible, medical management begins with alpha-adrenergic blockade (e.g., phenoxybenzamine), followed by beta-blockade to control reflex tachycardia [53]. Approximately 40% of pheochromocytomas are associated with germline mutations (e.g., MEN2, von Hippel-Lindau, NF1) [54]; thus, genetic counseling and testing are recommended upon diagnosis.

### 5.5 Cushing Syndrome 

Cushing syndrome, characterized by chronic cortisol excess, is associated with hypertension and increased cardiovascular risk [55, 56, 57]. Among patients with early-onset hypertension (<40 years), its prevalence ranges from 6.2% to 7.7% [58]. The duration of hypercortisolism correlates with the development of hypertension, emphasizing the importance of considering this etiology in young patients with difficult-to-control blood pressure [56, 57]. Cortisol excess promotes hypertension via upregulation of angiotensinogen, endothelin-1, enhanced mineralocorticoid activity, and increased sensitivity to sympathetic stimulation through beta-adrenergic receptors [56, 57, 58], contributing to vascular remodeling and elevated peripheral resistance [57]. Screening is recommended in individuals under 40 with grade 2 hypertension, those with sudden worsening of previously stable blood pressure, or in cases with absent nocturnal dipping on 24-hour monitoring. While treatment of hypercortisolism—often surgical—can improve blood pressure, remission is not always achieved. When pharmacologic therapy is needed, ACEi or ARBs are first-line due to their cardioprotective effects. If control is insufficient, calcium channel blockers (CCBs) and mineralocorticoid receptor antagonists (MRAs) may be added. Nitric oxide donors and alpha-blockers are reserved for resistant cases. Given the frequent presence of hypokalemia, thiazide diuretics should be used cautiously. MRAs are preferred in patients with concurrent hypertension and hypokalemia. Beta-blockers, particularly vasodilating agents, may be considered in those with prior myocardial infarction. Ultimately, treatment should be individualized due to the heterogeneous nature of the syndrome [58].

## 6. Screening for Hypertension-Induced Drugs

Several medications can contribute to RH and should be systematically reviewed. They contribute by exerting direct pressor effects or by interfering with the efficacy of antihypertensive agents. Table 1 (Ref. [3, 59, 60, 61, 62, 63, 64, 65, 66, 67]) summarizes common drugs associated with RH, their mechanisms of action, and recommended management strategies.

**Table 1.** **Common drug-induced mechanisms of hypertension and recommended interventions**.

Drug	Mechanism	Recommendation
Non-steroidal Anti-inflammatory Drugs (NSAIDs)	Inhibition of COX, leading to reduced prostacyclin synthesis, impaired vasodilation, and sodium retention [59].	Minimize or avoid systemic use when possible. Consider alternatives such as acetaminophen, tramadol, or topical NSAIDs, depending on clinical context [3].
Exogenous corticosteroids (glucocorticoids, mineralocorticoids)	Promote sodium and fluid retention, elevate cardiac output, and increase vascular tone [60].	Use the lowest effective dose or switch to non-systemic formulations (e.g., inhaled or topical) when feasible. Limit use if possible [3].
Decongestants and antitussives (pseudoephedrine, phenylephrine)	Contain sympathomimetic agents that induce systemic vasoconstriction [61].	Restrict use to short-term when necessary. Avoid in patients with uncontrolled or severe hypertension. Consider alternatives such as nasal saline, intranasal steroids, or antihistamines [3].
Hormonal therapies (combined oral contraceptives, hormonal replacement therapy)	Increase angiotensinogen levels and activate the renin-angiotensin system [62].	Use low-dose estrogen formulations (e.g., ≤30 mcg ethinyl estradiol), progestin-only pills, or non-hormonal contraceptives (e.g., barrier methods, IUDs). Avoid in women with poorly controlled hypertension [63].
Recreational drugs (cocaine, amphetamines)	Induce vasoconstriction and sympathetic overactivity [64]	Strongly advise cessation [3]. Refer for substance use treatment if appropriate.
Stimulants (methylphenidate, dextroamphetamine, modafinil, armodafinil, adrafinil)	Enhance sympathetic tone, leading to increased heart rate and vascular resistance [64].	Consider dose reduction or discontinuation. For ADHD, explore behavioral therapy or non-stimulant pharmacologic options [65].
Antineoplastic agents (VEGF inhibitors, e.g., bevacizumab; TKIs like sunitinib, sorafenib)	Causes endothelial dysfunction, vascular remodeling, and increased systemic resistance [66].	Monitor blood pressure closely. Start or adjust antihypertensive therapy as needed [3, 66].
Herbs/supplements: natural licorice, yohimbine	Licorice: inhibits 11β-HSD2, increasing cortisol’s effect on mineralocorticoid receptors [67].	Avoid use, especially in patients with or at risk for hypertension [3]. Screen supplement use routinely.

Abbreviations: COX, cyclooxygenase; IUD, intrauterine device; ADHD, attention-deficit/hyperactivity disorder; VEGF, vascular endothelial growth factor; TKI, tyrosine kinase inhibitor; 11β-HSD2, 11β-hydroxysteroid dehydrogenase type 2.

## 7. Current Treatments: Evidence-Based Approach

### 7.1 Lifestyle Intervention

The rising global prevalence of hypertension is strongly linked to Western lifestyle patterns, including sedentary behavior, physical inactivity, consumption of energy-dense foods, and excessive sodium intake [68]. These unhealthy habits are particularly common among patients with RH [69]. Lifestyle changes, such as regular physical activity, reduced protein intake or adherence to a Mediterranean diet, and avoidance of tobacco and alcohol, have been associated with improved blood pressure control and slower progression of CKD [70, 71]. However, few studies have specifically evaluated these interventions in RH populations, and small sample sizes limit generalizability [69]. Studies reported that adherence to two or more healthy lifestyle practices significantly reduced cardiovascular risk in RH patients [71]. One of the most rigorous investigations in this context is the TRIUMPH (Treating Resistant Hypertension Using Lifestyle Modification to Promote Health) trial. This single-center study evaluated the effects of structured interventions combining aerobic exercise, dietary counseling, and weight management in patients with RH. The average participant was 63 years old with a BMI of 36; approximately 24% had CKD. Compared to standard education, the “Center-Based Lifestyle Intervention” group achieved significant reductions in clinic and ambulatory systolic (>12 mmHg) and diastolic (>5 mmHg) blood pressures, along with a 14% increase in peak VO₂, longer exercise duration, and an average weight loss of 15.3 pounds. These findings underscore the efficacy of lifestyle interventions even in patients with pharmacologically treated RH [69].

### 7.2 Weight Management

Obesity is significantly associated with RH [72]. A body mass index (BMI) ≥25 kg/m^2^ also increases the risk of progression to end-stage renal disease (ESRD) [68]. Weight loss has been shown to reduce albuminuria and proteinuria, indicating a protective renal effect [68]. Proposed mechanisms linking obesity to RH include increased insulin resistance, chronic inflammation, sympathetic overactivity, activation of the RAAS, and elevated oxidative stress markers [71].

### 7.3 Diet

The DASH diet (Dietary Approaches to Stop Hypertension) has shown efficacy in lowering blood pressure in unmedicated hypertensive individuals; however, its effectiveness in patients with RH, where standard pharmacologic therapies have failed, remains uncertain [4]. Low-protein dietary patterns may help slow the progression of ESRD, and adherence to a Mediterranean diet has been associated with a lower risk of CKD progression [68].

### 7.4 Exercise

Physical inactivity is prevalent in approximately 40% of patients with RH [71]. Regular physical activity enhances cardiorespiratory fitness (CRF) and has demonstrated short- and long-term antihypertensive effects. For example, light to moderate-intensity aerobic exercise performed for approximately 45 minutes can reduce blood pressure for up to 5 hours post-exercise. Treadmill walking three times per week for 8–12 weeks has been associated with reductions in both office systolic and diastolic blood pressure. Among aerobic modalities, heated pool exercise appears particularly effective in lowering blood pressure [73]. A systematic review comparing exercise-based interventions (aerobic and dynamic resistance training) to placebo or first-line antihypertensive medications suggested a bias in favor of pharmacologic treatment; however, exercise showed meaningful benefits, particularly in low-risk hypertensive patients [70]. Overall, these findings underscore the importance of integrating structured physical activity into the management of RH as a complementary, nonpharmacologic strategy to optimize BP control.

### 7.5 Smoking and Alcohol

Tobacco use, primarily through nicotine exposure, is associated with acute increases in blood pressure and heart rate, driven by elevated cardiac output and peripheral vascular resistance [74]. Similarly, alcohol consumption has shown a dose-dependent relationship with the onset and progression of hypertension. These behaviors represent important, modifiable lifestyle factors, and their reduction or cessation is a key component of comprehensive blood pressure management, particularly in individuals with resistant hypertension [75].

## 8. Standard Pharmacologic Optimization

### 8.1 Triple Therapy

The cornerstone of antihypertensive pharmacologic treatment involves a combination of a RAAS inhibitor, a calcium channel blocker, and a diuretic. The diagnosis of RH requires that these three classes be prescribed at maximally tolerated doses [2]. Given the critical role of sodium balance in hypertension, appropriate diuretic selection is essential. Thiazide-like diuretics such as chlorthalidone and indapamide are preferred due to their longer half-life and more consistent 24-hour blood pressure control. Although some evidence suggests that chlorthalidone may provide more sustained blood pressure reduction than hydrochlorothiazide [2, 76], recent large trials have reported similar cardiovascular and safety outcomes between the two agents [77, 78]. Consistent with the 2025 AHA/ACC Guideline, patients with resistant hypertension should be switched from hydrochlorothiazide to chlorthalidone or indapamide when optimizing diuretic therapy [79].

Current guidelines consider ACE inhibitors and ARBs therapeutically equivalent, providing similar blood pressure reduction and cardiovascular protection. However, angiotensin II can still be generated through non-ACE pathways, particularly chymase, which may remain active despite ACE inhibition. This mechanism could contribute to persistent RAAS activation in a subset of patients [80]. Thus, switching from an ACE inhibitor to an ARB may be a physiologically reasonable individualized consideration in cases of apparent resistant hypertension, although this approach is not part of current guideline recommendations. Table 2 summarizes the efficacy, safety, and evidence grading for pharmacological and device-based therapies in resistant hypertension.

**Table 2.** **Efficacy, safety, and evidence grading for pharmacological and device-based therapies in resistant hypertension**.

Therapy	Efficacy	Safety/Common adverse events	Guideline recommendation (Class/Level)
ACE inhibitors/ARBs	Reduce SBP 8–10 mmHg, proven CV and renal protection	Cough (ACEi), hyperkalemia, angioedema	I/A
Thiazide-like diuretics	Sustained BP control, improved nocturnal BP	Hypokalemia, hyponatremia, hyperuricemia	I/A
Calcium channel blockers (dihydropyridines)	Reduce SBP 7–9 mmHg, effective in combination therapy.	Peripheral edema, flushing, headache	I/A
Mineralocorticoid receptor antagonists (spironolactone, eplerenone)	PATHWAY 2: Best fourth line agent, Reduce SBP 8–10 mmHg	Hyperkalemia, gynecomastia (spironolactone)	I/A (spironolactone); IIa/B (eplerenone)
Beta-blockers	Beneficial in CAD, HF, Arrhythmia, and modest BP effect	Bradycardia, fatigue, bronchospasm	IIa/B
SGLT2 inhibitors	Reduce SBP 4–6 mmHg, CV, and renal outcome benefit	Genital mycotic infections, volume depletion	IIa/B
GLP-1 receptor agonists	Reduce SBP 3–5 mmHg, improved glycemic control	Nausea, vomiting, gastrointestinal intolerance	IIb/B
Endothelin receptor antagonists (ERA)	Investigational, reduce BP 6–8 mmHg	Edema, hepatic enzyme elevation	IIb/C
ARNI (sacubitril/valsartan)	Reduce BP 5–7 mmHg, no formal HTN indication	Hypotension, hyperkalemia, angioedema	IIb/B
Renal denervation (RDN)	Reduce SBP 8–10 mmHg, suitable for resistant hypertension	Renal artery dissection, hematoma (<1%)	IIb/B
Baroreflex activation therapy (BAT)	Reduce SBP 10–15 mmHg, in uncontrolled resistant hypertension	Local infection, device pocket pain	IIb/C

Abbreviations: ACE, Angiotensin converting enzyme; ARB, Angiotensin receptor blocker; CV, Cardiovascular; SBP, Systolic blood pressure; BP, Blood pressure; CAD, Coronary artery disease; HF, Heart failure; ARNI, Angiotensin receptor and neprilysin inhibitor.

### 8.2 Diuretic Selection Based on Renal Function

Diuretic choice should be guided by renal function. Chlorthalidone remains effective at estimated glomerular filtration rates (eGFR) as low as 30 mL/min/1.73 m^2^, whereas hydrochlorothiazide is less effective below 45 mL/min/1.73 m^2^ [2, 76]. In patients with eGFR <30 mL/min/1.73 m^2^ or hypoalbuminemia, loop diuretics are preferred and may be used in combination with thiazides. Among loop diuretics, torsemide is favored due to its longer duration of action [2, 76].

### 8.3 Mineralocorticoid Receptor Antagonists (MRAs)

Spironolactone is the preferred fourth-line therapy for RH, as demonstrated in the PATHWAY-2 trial, where it showed superior BP-lowering efficacy compared to placebo, bisoprolol, and doxazosin (–8.7 mmHg vs. placebo; *p* < 0.0001) [81]. Its effectiveness appears proportional to baseline plasma renin activity, supporting the role of aldosterone-mediated volume overload in RH. However, adverse effects such as gynecomastia, menstrual irregularities, and hyperkalemia, particularly in CKD, can limit its use [82]. Eplerenone, a selective MRA with fewer endocrine side effects, represents an evidence-supported alternative for patients intolerant to spironolactone, although it may be less potent and often requires twice-daily dosing [83, 84]. Finerenone, a nonsteroidal MRA with anti-inflammatory and antifibrotic properties, has demonstrated cardiovascular and renal benefits in patients with diabetic kidney disease, but it is not approved for hypertension, and data in RH populations remain limited [85, 86].

In CKD, MRAs should be used with caution due to elevated hyperkalemia risk from impaired potassium excretion and concomitant RAAS blockade [87]. Mitigation strategies include lower dosing, preferring agents like finerenone, frequent monitoring of potassium and renal function, and consideration of potassium binders (e.g., patiromer, sodium zirconium cyclosilicate) in high-risk patients [88].

### 8.4 Beta Blockers 

Nebivolol is a third-generation β₁-selective blocker with nitric oxide–mediated vasodilatory effects. In contrast to traditional beta blockers, it enhances endothelial function and demonstrates neutral or favorable metabolic profiles, making it an appropriate choice for RH, particularly in patients with metabolic syndrome or obesity [89]. Its antihypertensive efficacy is comparable to that of ACEi and diuretics, with improved tolerability [90]. Carvedilol, a non-selective beta blocker with additional α₁-blocking properties, offers peripheral vasodilation and is especially beneficial in patients with RH and concurrent heart failure with reduced ejection fraction (HFrEF) or left ventricular hypertrophy [91]. Nonetheless, beta blockers may cause adverse effects such as fatigue, bradycardia, and impaired glycemic control in some individuals.

### 8.5 Central Sympatholytic Agents

Central α₂-agonists such as clonidine, methyldopa, and guanfacine lower blood pressure by reducing sympathetic outflow. Although effective, their use in RH is limited due to poor tolerability and side effects, including sedation, dry mouth, depression, and risk of rebound hypertension upon withdrawal [92]. These agents are typically reserved for short-term inpatient use or as a last resort in outpatient settings. Methyldopa remains a first-line option in pregnancy-induced hypertension but has limited utility in chronic RH outside this context [93].

Peripheral α₁-blockers, such as extended-release doxazosin, may provide modest benefit in RH, particularly in patients with coexisting benign prostatic hyperplasia (BPH). The extended-release formulation is preferred due to improved tolerability and a more stable antihypertensive effect [94]. However, their antihypertensive effect is inferior to agents like spironolactone [81], and their use is often restricted by adverse effects such as orthostatic hypotension and reflex tachycardia, especially in older adults.

### 8.6 Aliskiren and Dual RAAS Blockade

Aliskiren, a direct renin inhibitor, was initially promising because it targets the RAAS cascade at its origin and reduces angiotensin I generation. However, large clinical trials failed to demonstrate cardiovascular benefit, and its use has become restricted. While aliskiren can lower blood pressure, its role in RH is limited to highly selected cases under close monitoring due to increased risks of hyperkalemia, hypotension, and renal impairment, particularly in patients with diabetes or CKD [95]. Importantly, combining aliskiren with RAAS inhibitors (ACEi or ARB) or using dual RAAS blockade (ACEi + ARB) is not recommended and is generally contraindicated in clinical practice [96].

## 9. Emerging Therapies

### 9.1 Angiotensin Receptor and Neprilysin Inhibitor (ARNI)

Sacubitril/valsartan, an angiotensin receptor and neprilysin inhibitor (ARNI), has demonstrated antihypertensive efficacy, particularly in patients with heart failure. Compared to ACEi or ARBs, ARNI therapy may provide superior blood pressure reduction without increased adverse events. However, the heterogeneity of study populations limits the generalizability of current findings, and large-scale, long-term randomized controlled trials are needed to validate its role in RH [97].

### 9.2 Sodium–Glucose Cotransporter-2 Inhibitor (SGLT2i)

SGLT2 inhibitor (SGLT2i) have emerged as a promising adjunct in the management of RH, particularly in patients with coexisting diabetes or CKD. While originally approved for glycemic control and HF, these agents have shown consistent reductions in blood pressure, with mean decreases of 3–6 mmHg systolic and 1–2 mmHg diastolic [98, 99]. The antihypertensive effect of SGLT2i is thought to result from mild natriuresis, reduced plasma volume, and modulation of neurohormonal pathways, including inhibition of the renin-angiotensin-aldosterone system and sympathetic nervous system [2, 99]. In patients with RH, SGLT2i have demonstrated meaningful reductions in both office and ambulatory blood pressure, even when added to optimized antihypertensive regimens [100]. Notably, a substantial proportion of patients achieved target systolic BP <130 mmHg, reinforcing their potential role as an adjunctive therapy in difficult-to-control hypertension [100]. Beyond blood pressure, SGLT2i have also been associated with reduced cardiovascular mortality, heart failure hospitalizations, and progression of nephropathy in RH populations. Their renal benefits, including reduced intraglomerular pressure and albuminuria, further support their use in RH patients with CKD, even in the absence of albuminuria. These effects occur early after initiation and are largely independent of glycemic control. SGLT2i are well tolerated, with a favorable safety profile, making them an attractive option in selected RH patients [99].

### 9.3 GLP-1 Receptor Agonists (GLP-1 RA)

GLP-1 receptor agonists (GLP-1 RA), originally developed for glycemic control in type 2 diabetes mellitus, have demonstrated multiple cardiometabolic benefits that make them attractive adjuncts in the management of RH. These agents exert favorable effects on weight reduction, appetite regulation, and inflammation, and have shown antihypertensive potential, particularly in patients with coexisting obesity, diabetes, or CKD [101]. GLP-1 RA lowers blood pressure modestly, with average systolic reductions of 2–5 mmHg, an effect that appears to be independent of their glucose-lowering properties. The mechanism may involve natriuresis, reduced sympathetic activity, weight loss, and improved endothelial function [101]. Their use is especially relevant in RH populations where metabolic syndrome and obesity are common contributors to poor blood pressure control. Evidence from cardiovascular outcomes trials such as LEADER, SUSTAIN-6, and REWIND has highlighted the cardioprotective and renoprotective effects of GLP-1 RA, including reduced progression of albuminuria and preservation of eGFR [101]. These findings support their consideration in RH patients with high cardiovascular or renal risk. In addition, GLP-1 RA has demonstrated significant weight loss effects, as observed in the SELECT trial, offering further benefits in patients with obesity-related hypertension [102]. However, these agents are contraindicated in individuals with a personal or family history of medullary thyroid carcinoma or multiple endocrine neoplasia syndrome type 2, and may cause gastrointestinal side effects and, rarely, pancreatitis [103]. Their safety during pregnancy has not been established.

### 9.4 Renal Denervation

Renal denervation has emerged as a promising non-pharmacological option for patients with RH, a population at high cardiovascular risk despite adherence to multiple antihypertensive medications. In addition to suboptimal blood pressure control, poor treatment adherence and the burden of polypharmacy highlight the need for alternative therapeutic approaches [4, 104]. Catheter-based renal sympathetic denervation, performed via radiofrequency or ultrasound ablation, has shown efficacy in lowering blood pressure in both resistant and non-resistant hypertensive populations. The SYMPLICITY HTN-3 trial demonstrated the safety of the procedure [105], while the SPYRAL HTN-OFF MED trial confirmed that renal denervation significantly reduced blood pressure compared with sham procedures, even in the absence of pharmacologic therapy [106]. The SPYRAL HTN-ON MED trial further showed that the blood pressure–lowering effect was sustained over 36 months, independent of medication adjustments and without major safety concerns [107]. Renal denervation represents a valuable adjunctive therapy in RH, particularly for patients with poor adherence or inadequate response to maximal pharmacologic treatment.

### 9.5 Baroreflex Activation Therapy

Baroreflex activation therapy (BAT), also known as cardiac neuromodulation therapy, is a device-based intervention designed to reduce sympathetic tone and lower blood pressure. It modulates the autonomic nervous system by delivering electrical stimulation through a pacemaker-like device, thereby inhibiting baroreceptor-mediated sympathetic activation [108]. This approach has demonstrated sustained reductions in both office and 24-hour ambulatory systolic blood pressure. In the MODERATO II trial, patients experienced over 10 mmHg reductions in ambulatory systolic blood pressure and over 15 mmHg in office systolic blood pressure at long-term follow-up [109]. BAT may be particularly relevant in patients with RH who already require permanent pacing, offering blood pressure control without the need for additional medications or invasive procedures.

## 10. Investigational Agents

### 10.1 Endothelin Receptor Antagonist: Aprocitentan and Bosentan

Endothelin-1 (ET-1) is a potent vasoconstrictor upregulated in patients with RH. Aprocitentan, a dual endothelin receptor antagonist (ERA), demonstrated significant blood pressure reduction in the phase 3 PRECISION trial. Among patients with RH, aprocitentan 25 mg daily reduced systolic BP by 3.8 mmHg versus placebo during the withdrawal phase and maintained durable efficacy for up to 40 weeks, with fluid retention as the most frequent adverse effect [110]. Bosentan, an earlier dual ERA, showed antihypertensive properties in prior studies but is limited by hepatotoxicity and the need for frequent liver function monitoring [111].

### 10.2 Aldosterone Synthase Inhibitors: Baxdrostat and Lorundrostat

Aldosterone synthase inhibitors (ASIs) such as baxdrostat and lorundrostat suppress aldosterone production by inhibiting CYP11B2, offering a targeted alternative to mineralocorticoid receptor antagonists. In the phase 3 LAUNCH-HTN trial, lorundrostat achieved a placebo-adjusted systolic BP reduction of 9.1 mmHg at 6 weeks [112, 113]. The treatment was generally well tolerated, although mild hyperkalemia and transient declines in eGFR were observed. Baxdrostat has shown promising results in early trials, with a favorable safety profile [112].

### 10.3 Small Interfering RNA (siRNA) for Angiotensinogen: Zilebesiran

Zilebesiran is a GalNAc-conjugated small interfering RNA (siRNA) that silences hepatic angiotensinogen synthesis, and is a subcutaneous injection that reduces RAAS activity upstream of renin. In the KARDIA-1 phase 2 trial, a single dose of ≥200 mg reduced ambulatory systolic BP by up to 16.7 mmHg at 3 months, with effects lasting up to 6 months [113, 114, 115, 116]. The most common adverse events were mild injection site reactions and transient hyperkalemia. Phase 1 data confirmed >90% suppression of serum angiotensinogen without inducing hypotension, renal dysfunction, or severe electrolyte disturbances [117]. Long-acting dosing and minimal adherence burden make zilebesiran an attractive investigational agent in RH. A reversal agent (REVERSIR) is in development for added safety in acute settings [115].

### 10.4 Gene and Cell Therapies

Although hypertension is a multifactorial disease, gene and cell-based approaches are under investigation. Preclinical strategies include overexpression of vasodilatory genes (e.g., endothelial nitric oxide synthase, kallikrein) and silencing of vasoconstrictive pathways such as RAAS components, both of which have reduced BP in animal models [114, 118, 119]. Additionally, cell-based therapies, such as engineered endothelial progenitor cells, aim to restore vascular function and reduce inflammation [120, 121]. While still experimental, these approaches may offer future therapeutic options for durable BP control in RH.

## 11. Proposed Therapeutic Algorithms and Clinical Guidelines

Major cardiovascular and nephrology societies have developed frameworks for the management of RH, with general agreement on diagnostic and treatment principles. However, differences exist in the timing of updates and certain therapeutic recommendations. The most recent guidelines include those from the European Society of Cardiology (ESC, 2024), the American College of Cardiology/American Heart Association (ACC/AHA, 2017), the International Society of Hypertension (ISH, 2020), and Kidney Disease: Improving Global Outcomes (KDIGO, 2021) [3, 11, 114, 122]. All four organizations emphasize a structured initial evaluation: confirm elevated blood pressure, exclude pseudoresistance (e.g., nonadherence, white coat effect), and assess for secondary causes. Non-pharmacologic interventions—particularly sodium restriction, weight loss, and lifestyle changes—are uniformly recommended before intensifying pharmacologic therapy.

There is a strong consensus on the use of a mineralocorticoid receptor antagonist as fourth-line therapy in RH. KDIGO cautions about the elevated risk of hyperkalemia in patients with CKD, while ESC 2024 recommends eplerenone as an alternative in those intolerant to spironolactone. Notably, ESC is the only society to explicitly recommend beta-blockers as fifth-line agents. Other guidelines mention additional classes (e.g., alpha-blockers, central agents) without prioritization. ESC 2024 is also the first to endorse interventional therapies, such as renal denervation, within a shared decision-making framework, recommending restriction to experienced centers. In contrast, ACC/AHA 2017 did not endorse device-based interventions due to insufficient evidence at the time, and neither KDIGO nor ISH addresses these approaches. However, a 2023 scientific statement from the AHA acknowledged renal denervation as a potential future option in selected patients following FDA approval [123].

**Fig. 3.**
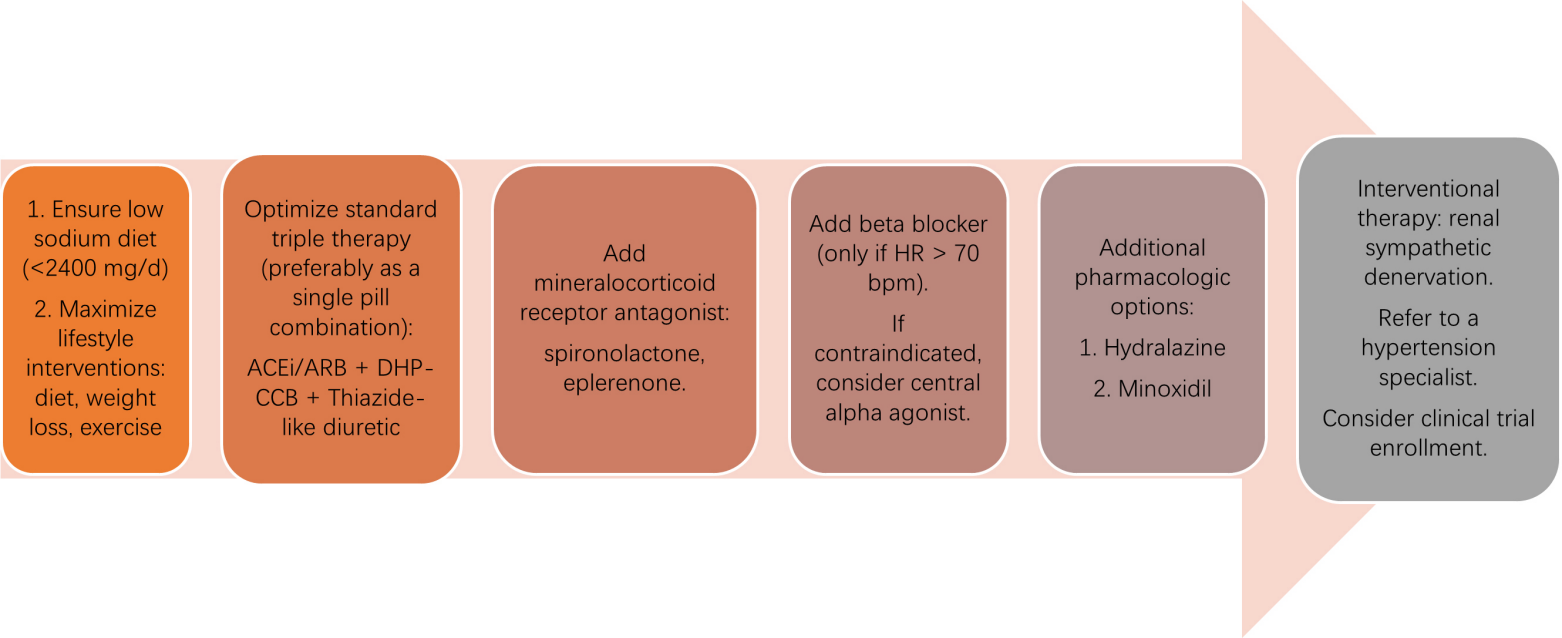
**Stepwise pharmacologic and interventional management of resistant hypertension**. The diagram outlines a sequential approach to treating resistant hypertension, beginning with lifestyle optimization and sodium restriction, followed by standard triple therapy with an ACE inhibitor (ACEi) or angiotensin receptor blocker (ARB), a dihydropyridine calcium channel blocker (DHP-CCB), and a thiazide-like diuretic. If blood pressure remains uncontrolled, a mineralocorticoid receptor antagonist (spironolactone or eplerenone) is added, followed by a β-blocker when heart rate (HR) is >70 bpm or, if contraindicated, a central α-agonist. Additional options include hydralazine or minoxidil. For patients with persistent hypertension despite these therapies, referral to a hypertension specialist, evaluation for clinical trial enrollment, or consideration of renal sympathetic denervation is recommended. © 2025 “Used with permission of Mayo Foundation for Medical Education and Research, all rights reserved.”

Referral to specialized hypertension centers is consistently supported. ESC advises referral once three medications are required; ACC/AHA suggests reassessment after six months of optimized four-drug therapy. ISH does not specify thresholds but emphasizes the benefits of dedicated multidisciplinary care. In clinical practice, referral to a Hypertension Clinic or Hypertension specialist is appropriate when patients remain uncontrolled despite optimized triple therapy, when secondary causes are suspected, when medication intolerance limits escalation, or when considering device-based interventions such as renal denervation (Fig. 3).

The ESC 2024 guidelines also highlight emerging therapies—including SGLT2 inhibitors, sacubitril/valsartan, and dual endothelin receptor antagonists—as promising future options in RH. However, these agents were not yet integrated into formal algorithms due to the limited availability of outcome data at the time of publication. The forthcoming 2025 ACC/AHA guidelines, expected in September, may clarify how novel therapies will be incorporated into RH management [124].

## 12. Future Perspectives and Research Areas

The management of RH is shifting toward precision medicine, emphasizing individualized approaches based on pathophysiologic profiling. Rather than applying uniform treatment algorithms, emerging strategies aim to identify specific mechanisms—such as sympathetic overactivity, intravascular volume expansion, or RAAS hyperactivation—to guide therapy selection [125, 126]. Tools such as biochemical markers, hemodynamic measurements, and wearable sensors are under investigation for their potential to stratify patients and optimize therapeutic responses [125].

Artificial intelligence (AI) is also transforming hypertension care. Machine learning algorithms can synthesize data from electronic health records, medication adherence patterns, and home BP monitoring to identify patients at risk for poor control or adverse outcomes [127]. These tools can assist in detecting white coat hypertension, predicting treatment response, and identifying nonadherence, thereby enabling more targeted interventions. When integrated with remote monitoring systems, AI can support real-time clinical decision-making and long-term management in RH [128, 129, 130].

Despite these advances, challenges persist. Biomarker-based and AI-driven models require further validation in diverse populations, and issues of data privacy, equity, and algorithmic transparency must be addressed. Nonetheless, the integration of digital health technologies, multi-omic profiling, and AI holds significant promise for transitioning RH management toward a more precise, proactive, and patient-centered paradigm [126, 127].

## 13. Limitations

This review is narrative rather than systematic; therefore, publication bias and heterogeneity among studies cannot be excluded. Most evidence on resistant hypertension originates from observational or single-center trials, and emerging strategies, including AI-driven models and endotype-based classifications, remain in early phases of research. These approaches require multicenter validation before clinical adoption. Furthermore, the long-term safety and comparative efficacy of device-based interventions are still being investigated.

## 14. Conclusions

RH represents a complex and high-risk phenotype of blood pressure dysregulation, associated with increased cardiovascular and renal morbidity. Accurate diagnosis requires a structured evaluation to exclude pseudoresistance, confirm medication adherence, and identify secondary causes. While standard treatment relies on optimized triple therapy and the addition of mineralocorticoid receptor antagonists, new pharmacologic agents, such as SGLT2 inhibitors, GLP-1 receptor agonists, and endothelin receptor antagonists, are expanding therapeutic options for selected patients. Device-based interventions, particularly renal denervation, are gaining recognition within updated clinical guidelines and may offer durable blood pressure reduction in carefully selected individuals. Emerging investigational therapies, including aldosterone synthase inhibitors and RNA-based technologies, reflect a promising shift toward mechanism-based approaches. Looking ahead, precision medicine, artificial intelligence, and digital health technologies are poised to transform RH management, enabling real-time, individualized care. However, widespread adoption will require further validation, infrastructure development, and ethical oversight. As clinical guidelines evolve and novel therapies mature, a multidisciplinary, patient-centered strategy remains essential for optimizing outcomes in this challenging population.

## Abbreviations

ABPM, ambulatory blood pressure monitoring; ACEi, angiotensin-converting enzyme inhibitor; AI, artificial intelligence; ARB, angiotensin receptor blocker; ARNI, angiotensin receptor and neprilysin inhibitor; aSBP, ambulatory systolic blood pressure; BAT, baroreflex activation therapy; BP, blood pressure; BPH, benign prostatic hyperplasia; CKD, chronic kidney disease; CNT, cardiac neuromodulation therapy; CRF, cardiorespiratory fitness; CTA, computed tomographic angiography; eGFR, estimated glomerular filtration rate; ERA, endothelin receptor antagonist; ESC, European Society of Cardiology; GLP-1 RA, glucagon-like peptide-1 receptor agonist; HF, heart failure; ISH, International Society of Hypertension; KDIGO, Kidney Disease: Improving Global Outcomes; MRA, mineralocorticoid receptor antagonist; PCT, proximal convoluted tubule; RAAS, renin–angiotensin–aldosterone system; RH, resistant hypertension; siRNA, small interfering RNA; SGLT2i, sodium-glucose cotransporter-2 inhibitor.
